# European health regulations reduce registry-based research

**DOI:** 10.1186/s12961-024-01228-1

**Published:** 2024-09-30

**Authors:** Oscar Brück, Enni Sanmark, Ville Ponkilainen, Alexander Bützow, Aleksi Reito, Joonas H. Kauppila, Ilari Kuitunen

**Affiliations:** 1 Hematoscope Lab, Comprehensive Cancer Center & Department of Clinical Chemistry, Diagnostic Center, Helsinki University Hospital & University of Helsinki, Biomedicum I, Haartmaninkatu 8, P.O. Box 700, 00290 Helsinki, Finland; 2 Department of Otorhinolaryngology and Phoniatrics-Head and Neck Surgery, Helsinki University Hospital, Finland and Faculty of Medicine, University of Helsinki, Helsinki, Finland; 3 Center for Musculoskeletal Diseases, Tampere University Hospital, Tampere, Finland; 4 Krogerus Attorneys Ltd, Helsinki, Finland; 5 Department of Surgery, Oulu University Hospital, University of Oulu, Oulu, Finland; 6 Department of Molecular Medicine and Surgery, Karolinska Institutet, Karolinska University Hospital, Stockholm, Sweden; 7 Department of Pediatrics, Institute of Clinical Medicine, University of Eastern Finland and Kuopio University Hospital, Kuopio, Finland

## Abstract

**Background:**

The European Health Data Space (EHDS) regulation has been proposed to harmonize health data processing. Given its parallels with the Act on Secondary Use of Health and Social Data (Secondary Use Act) implemented in Finland in 2020, this study examines the consequences of heightened privacy constraints on registry-based medical research.

**Methods:**

We collected study permit counts approved by university hospitals in Finland in 2014–2023 and the data authority Findata in 2020‒2023. The changes in the study permit counts were analysed before and after the implementation of the General Data Protection Regulation (GDPR) and the Secondary Use Act. By fitting a linear regression model, we estimated the deficit in study counts following the Secondary Use Act.

**Results:**

Between 2020 and 2023, a median of 5.5% fewer data permits were approved annually by Finnish university hospitals. On the basis of linear regression modelling, we estimated a reduction of 46.9% in new data permits nationally in 2023 compared with the expected count. Similar changes were neither observed after the implementation of the GDPR nor in permit counts of other medical research types, confirming that the deficit was caused by the Secondary Use Act.

**Conclusions:**

This study highlights concerns related to data privacy laws for registry-based medical research and future patient care.

## Key Points

- Given its parallels with the European Health Data Space regulation, we modelled its possible consequences for registry-based medical research using data from Finland, where the Act on the Secondary Use of Health and Social Data (Secondary Use Act) has been implemented since 2020.
- Adjusted for the historical trend of increasing registry-based research conducted in Finnish university hospitals, the data permit count was almost 50% lower than expected in 2023.
- Similar changes were not observed post-GDPR or in other medical research types.
- This study demonstrates how increased data privacy regulations might reduce medical research, innovations and advances in future patient care.

## Introduction

Registry-based medical research forms an integral pillar of modern healthcare. Medical registries are invaluable resources for generating evidence-based medical guidelines and developing innovations such as effective diagnostic and monitoring tools. However, this progress has been subjected to a growing regulatory burden in the European Union (EU), particularly in the realm of data privacy and security, which are also part of the Charter of Fundamental Rights of the EU. The General Data Protection Regulation (GDPR), implemented in 2018, has strengthened personal data protection rights by providing individuals with greater control over their data and imposing stricter obligations on data controllers and processors. The European Health Data Space (EHDS) has been recently approved for establishing a common framework for the governance and sharing of health data across the European Union. While this initiative holds promise for facilitating cross-border access to health data and common policies for data privacy in health research, it also introduces additional layers of bureaucracy and potential delays in research and innovation, raising questions of its effectiveness [1].

The GDPR, as an EU regulation, is directly applicable to all member states, as will be the case with the EHDS. However, prior to the applicability of the EHDS, registry-based medical research was governed by member state law. In Finland, the legislation governing registry-based medical research consists primarily of the following: the Data Protection Act (1050/2018), and more importantly, the Act on the Secondary Use of Health and Social Data (552/2019; Secondary Use Act).

**Fig. 1:**
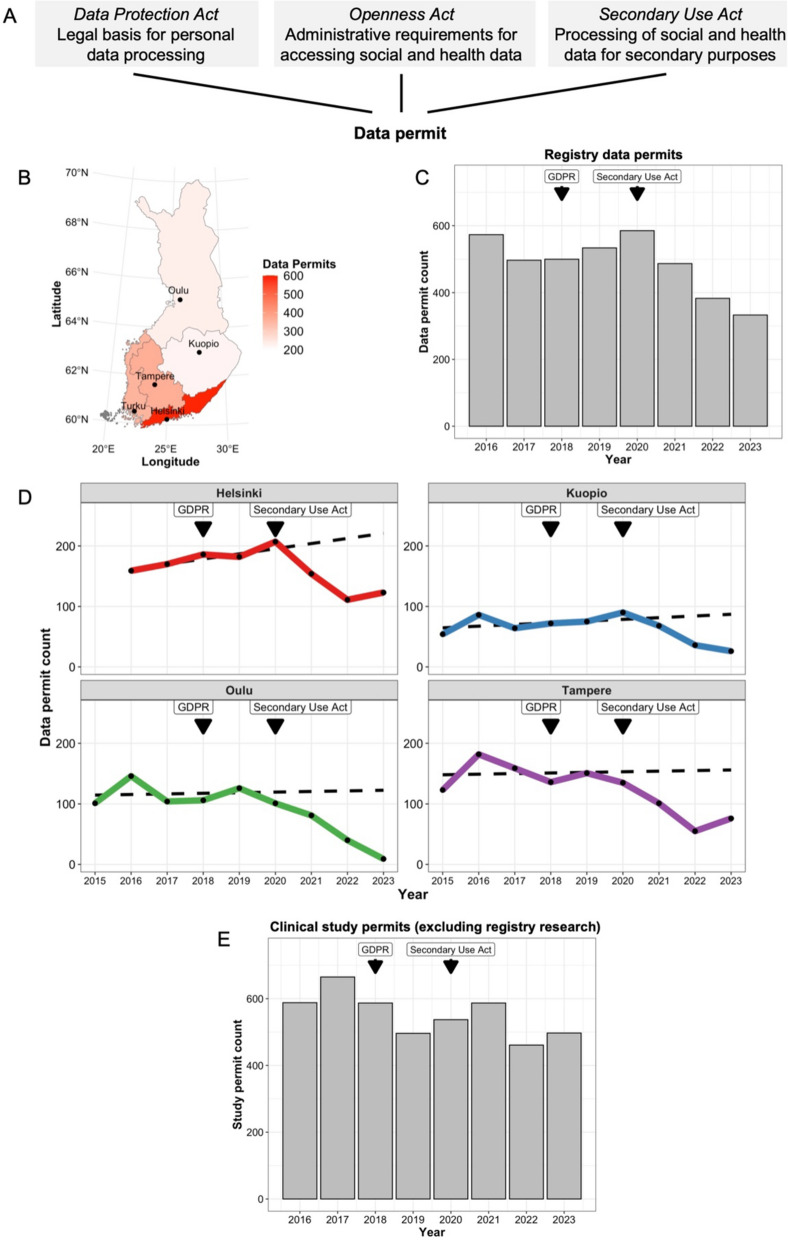
Registry-based research in Finland. **A** Overview of the essential regulations relevant for applying a registry-based medical research data permit in Finland. The Data Protection Act is a Finnish supplement to the General Data Protection Regulation. Abbreviations: Act on the Openness of Government Activities, Openness Act; Act on the Secondary Use of Health and Social Data, Secondary Use Act. **B** Map of Finland demonstrating cities (points) with a university hospital and their associated healthcare regions. **C** Bar plot of total data permit counts for registry-based research approved by university hospitals. Bars after 2020 include also the permits approved by Findata and these are multiplied by the number of university hospital registries these cover to ensure comparability. **D** Line plots of median counts of approved registry data permits by distinct Finnish university hospitals in 2015–2023. These are accompanied by fitted regression curves predicting permit counts on the basis of trends before the Secondary Use Act in 2015–2019 (dashed lines). **E** Bar plot of total data permit counts for clinical research excluding registry-based research approved by university hospitals

The Data Protection Act is a Finnish supplement to the GDPR (Fig. 1A). It parallels the Federal Data Protection Act (BDSG) in Germany, the Data Protection Act (Loi Informatique et Libertés) in France and many other national legislations implemented by member states. The Data Protection Act in Finland specifies that the public interest under point (e) of Article 6(1) GDPR is a valid legal basis for processing personal data for the purpose of scientific research. The processing of special categories of personal data, such as health data and genetic data, is also permitted but subjected to appropriate safeguards as further specified in the Act and as required under Article 89(1) GDPR. This act is crucial for registry-based medical research, as, together with the GDPR, it defines the core data protection framework under which health data are processed in Finnish scientific research. However, it does not specify how registry data are obtained for such purposes.

The Finnish national legislative model in this instance focusses primarily on controlling who may have access to sensitive data and under what circumstances, such as by regulating the relevant permit and consent processes and the relevant documentary requirements. This is largely the result of the relevant lex generalis, the Act on the Openness of Government Activities (21.5.1999/621; Openness Act). This approach is conceptually very different from the model of EU data protection legislation, which is largely agnostic as to how personal data are obtained but instead focusses on regulating the planned or ongoing data processing activities themselves. As a result, Finnish registry-based research is regulated under two different legislative paradigms with different targets.

Prior to the Secondary Use Act, registry-based medical research involving medical data of a single public health care entity required a permit granted by that entity as set out in i.a. Section 28 of the Openness Act. As such, studies could be combined with interventions related to the personal integrity of study participants, such as the collection of biological samples, which requires prior ethical approval, and administrative processes could be combined into a single permitting process. This process resulted in a study permit, which was granted by the relevant public healthcare entity. If registry data were required from multiple registries, the Finnish Institute for Health and Welfare acted as the permit authority for the relevant registry data.

The Ministry of Social Affairs and Health introduced the Secondary Use Act in Finland, which entered into force in 2019. Its primary purpose was to facilitate the effective and safe processing of personal social and health data for secondary uses, such as research, policymaking and healthcare management, in contrast to primary uses, such as the provision of healthcare services. Individual public health care entities, such as university hospitals, retained the right to grant data permits to their own data in single-registry studies. In contrast, Findata was established as the centralized data permit authority, that is, a one-stop agency granting data permits for secondary use, granting data permits for private healthcare providers, the National Patient Data Repository, or a combination of multiple social and health registries, with multiple other tasks, such as defining policies for safe data processing and assessing the anonymity of data. With the exception of the Health Data Hub in France and Denmark Statistics in Denmark, the scope and extent of the responsibilities are internationally unique.

Despite these positive objectives, two independent surveys conducted in 2021 highlighted the discontent of the research community with the Secondary Use Act. The first study focussed on medical doctors and revealed that, out of 430 responders, 79% experienced the data permit process to have become more complex than before, 55% that research projects were delayed and 64% that research costs had increased [2]. Owing to these issues, 42% of the responders reported that they had not initiated a study [2]. Similar results were reported in a second survey commissioned by the Ministry of Social Affairs and Health, where responders (*n* = 260) reported that the Secondary Use Act simplified neither the application process nor the combination of multiple registries [3].

Compared with prior practices, the Secondary Use Act contains very specific provisions on documentation requirements; how registry data are collected, combined, transferred and processed prior to their disclosure to the study that has received the data permit; the costs associated with such provisions; and the regulation of secure cloud-based computing instances in which the recipient project may conduct research on such data. In cases where Findata is the authority granting the data permit, researchers of the registry-based study cannot actively participate in the pre-transfer data processing activities. In such cases, the collection and processing work is conducted by the registry authorities and Findata, which may obligate the researchers to both longer processing times and costs related to data collection and processing.

While the EHDS regulation is expected to harmonize the principles of health data processing in the EU, there is little retrospective evidence on the impact of increasing privacy regulations on registry-based social and medical research. Given its considerable overlap with the Secondary Use Act in terms of shared objectives, implementation of national data permit authorities and establishing audited data secure environments, we sought to evaluate the impact of the regulation on registry-based research. Here, we examine data permit counts before and after the implementation of the act to reflect the administrative and procedural hurdles faced by researchers, providing a simple measure of how regulatory changes affect access to health data for research purposes.

## Methods

### Research registries

Population-based, healthcare and social registries represent retrospective research registries as information has been collected for another primary purpose but may be subject to secondary use in research registries. In contrast, in cohort studies in which subjects are first consented to a medical study, data are recorded and stored longitudinally for their primary use, representing prospective research registries. Retrospective registries have become increasingly popular in Finland due to their extent, availability of high-quality information in digital format and low cost, as these are maintained and managed by organizations or the public sector.

### Study versus data permits

There are five university hospitals in Finland, which are located in Helsinki, Tampere, Kuopio, Turku and Oulu. When we reference these cities, we are specifically alluding to the respective university hospitals. University hospitals serve as regional hubs for specialized care and provide comprehensive medical services to their respective catchment areas. Hospitals are well equipped for registry-based research because of their advanced IT infrastructure and larger patient populations, enabling them to collect, manage and analyse large-scale health data effectively both for primary and secondary purposes.

After entry into force of the Secondary Use Act, both a study permit and a data permit are required to conduct registry-based research projects in Finland. Prior to this, only study permits were needed, as explained above. The study permit is issued by the research organization, for example, the university hospital, where the research project is based. This ensures that the research is scientifically justified and ethical and that the rights of the participants are protected, although registry research permits are rarely handled in separate ethical research boards. The data permit is issued by either Findata or GDPR-defined data controllers (e.g. university hospitals or primary healthcare registries) whose registry data are used in the research to evaluate the purpose of data use, the security measures in place and the potential impact on individual privacy. While this dual-permit system ensures that both the data and the study are subject to rigorous scrutiny, data privacy measures have been an integral part of the study permit process before the Secondary Use Act. For simplicity, in this study, we employ the term data permit, as university hospitals can grant both study and data permits, but Findata can grant only data permits.

### Data collection

In January 2024, we solicited counts of new data permits for registry-based research, specifically involving university hospital registries, from the research departments of all five university hospitals (Helsinki, Turku, Tampere, Oulu and Kuopio). To account for variations in research funding and regulatory changes, we also requested data on study permits for medical research involving subjects, human tissue or medical devices. Owing to varying archiving practices, data from 2016 to 2023 were available from Tampere, Oulu, Kuopio and Helsinki. As data were accessible only from 2020 to 2023, Turku was excluded from analyses requiring data prior to 2020.

We also solicited counts of new data permits (*n* = 375) involving clinical data from university hospitals and grants from Findata from 2020 to 2023. Recognizing that Findata-approved permits cover data from 1–5 hospitals, we integrated details on the annual mean count of university hospitals covered by these permits (mean 2.2–2.6 from 2021 to 2023).

### Statistical analysis

We fitted univariate linear regression analyses using data permit counts as covariate and year as a predictor and examined the slopes of the curves by extracting the coefficient. We used the Mann‒Kendall test to estimate trend over time. On the basis of prior findings, we tested only whether the registry-based study count had decreased since its implementation with the one-sided Mann‒Kendall test [2, 3]. Elsewhere, we applied two-sided tests. We performed statistical analyses and visualizations with R 4.0 via the packages base, sf, mapsFinland and ggplot2.

## Results

In 2020–2023, 1768 registry-based research data permits were granted by university hospitals (Fig. 1B). Most of these (*n* = 595) were approved by Helsinki, followed by Tampere (*n* = 367), Turku (*n* = 355), Oulu (*n* = 231) and Kuopio (*n* = 220). Following a stable period between 2016 and 2019 (median 517, range 497–573; Fig. 1C), new data permit counts decreased rapidly across hospitals (tau −1, *p* = 0.042, one-sided Mann‒Kendall test). Compared with 2019, there was a median decrease of 22.4 (range 9.7–31.7) data permits per year, representing an annual median reduction of 5.5% (range 3.4–10.5%). At the same time, 375 registry-based data permits were approved by Findata (Fig. 1C). However, the percentage of Findata-approved data permits also decreased from 141 to 99 (29.8%) in 2023 (Fig. 1C). The initial increase could be partly explained by an accumulation of applications due to new systems and policies being established at Findata during 2020–2022. The following decrease in 2023 could hence reflect the pruning of the backlog.

Next, we examined data permit counts approved by university hospitals before and after (2016–2017 versus 2018–2019) the implementation of the GDPR. An annual median of 152 new registry research permits were approved pre-GDPR (Fig. 1C). While the corresponding median in the following 2 years was 131, the count increased from 121 in 2018 to 138 permits in 2019, implying that the GDPR had no conclusive impact on data permit counts (Fig. 1C).

To estimate the decline in data permits, we examined the era before the implementation of the Secondary Use Act. In 2015–2019, a median of 1.9 (range 1.0–7.3) additional data permits were approved, corresponding to a 0.70% (range 0.20–1.3%) yearly accumulation. The finding demonstrates that new permits approved for registry-based research remained stable prior to the implementation of the Secondary Use Act. Assuming that similar progress would have continued until 2023, we fitted a linear regression curve for each hospital and predicted that a total of 586.8 new data permits would have been granted in 2023 (Fig. 1D). The figure contrasts with the 234 new data permits actually approved. Given that a portion of multi-centre permits have been directed to Findata and that Turku was excluded from the analyses due to missing data, we included data permits accorded by Findata in 2023 (*n* = 99) multiplied by 78.3%, reflecting the percentage of data permits approved by university hospitals other than Turku in 2023. The estimated reduction was 275.3 [= 586.8 − 234.0 − (99.0 × 0.78)] data permits, corresponding to a relative reduction of 314.2/586.8 = 46.9%.

To exclude other confounding factors, we examined the data permit counts for other types of medical research (Fig. 1E). While the proportion of approved permits decreased in 2016–2023 (tau −0.54, *p* = 0.061, two-sided Mann‒Kendall test), the changes did not coincide with either the enactment of the GDPR or the Secondary Use Act.

## Discussion

This retrospective observational cohort study demonstrated the negative impact of the Secondary Use Act on registry-based studies only 3 years after its implementation. The findings sharply contrast with the generally positive attitude towards the secondary use of personal data in medical research [4].

This study has several implications for efforts to understand the potential impact of EHDS regulation on research and innovations. First, while research with retrospective registries could be previously performed with a minimal budget, solid funding has become necessary following the enactment of the Secondary Use Act. Data collection and application fees for submitting a study plan, adding researchers to a valid permit and processing data in secure cloud-based computing instances accumulate significant research costs.

Second, stringent data privacy laws may hinder individual countries from participating in multicenter registry studies and potentially impede global health by slowing responses to pandemics, such as coronavirus disease 2019 (COVID-19) [5]. Finland has a long tradition of accessing nationwide healthcare registries, such as the care registry for specialized healthcare, the cancer registry and implant registries, which have all been acknowledged for their coverage and quality [6–8]. Integrating Finnish registry holders in international studies is possible only if data are processed in a computing environment that has been audited to meet the legal requirements. Currently, only nine such environments are eligible, and all are based in Finland, implying that international registry studies would require the transfer of all data to one of these environments [9].

The main source of error in this study stems from the prediction of new data permits in 2023 in a scenario without the Secondary Use Act. However, we anticipate that registry-based research would have gained even more popularity than before, given the substantial investments in healthcare information technology infrastructures facilitating the release of electronic health records to cloud-based computing instances. Thus, many researchers have reported that the regulatory environment hampers not only conventional registry-based studies, but also promising progress towards automatic computer-assisted data curation and disease phenotyping [2, 10].

The enactment of the Secondary Use Act coincided with the COVID-19 pandemic, which could confound the results. Multiple reports have indicated an increase in scientific publishing following the COVID-19 outbreak [11, 12]. The increase in approved non-registry permit counts in 2020–2022 parallels with the increase in approved registry-based research permits in 2020, suggesting that the pandemic likely would not have reduced research activity. However, the exact influence of the pandemic on registry-based research would require examining study permit counts in other countries.

In addition, it is plausible that increasing bureaucratic requirements would have motivated researchers to apply for fewer and larger study and data permits to avoid administrative burden related to preparing applications. However, this speculation can be investigated in the future by comparing the number of publications per permit by their year of approval.

The enactment of the Secondary Use Act is grounded in the need to balance data accessibility with privacy protection, which is an uncompromised requirement of professional and ethical data use. This balance aims to safeguard personal information while promoting scientific advancements. However, the mechanisms by which this legislation operates can inadvertently stifle research. Even if a registry-based study is fully GDPR compliant, this does not guarantee that it is possible to conduct the study under the Secondary Use Act. As noted, the Secondary Use Act and the proposal for the EHDS exhaustively regulate the processing procedures both prior to and after the transfer of health-registry data for use in the research project. These procedures are largely mandatory and one-size-fits-all in nature, thus not accounting for the specific risk profile of the research project in question or the registry data involved, as would be the approach warranted by the GDPR and as was the case in Finland prior to the entry into force of the Secondary Use Act. This inflexibility and the resulting administrative hurdles delay research timelines and inflate costs. We posit that the specific inflexible processing and procedural requirements and the associated costs resulting from the Secondary Use Act are reasons for the reduction in data permits. These regulatory demands, although designed to enhance data protection and data security, may thus paradoxically hinder progress in medical research and patient care.

In conclusion, the results emphasize the need to balance effective and secure data research, that is, protecting the privacy of research subjects and providing research results benefitting the society as a whole. Medical researchers should be involved in planning, interpreting and assessing health data regulations. In addition to their complexity, the cumulative effect of European and national regulations has created a challenging environment for medical researchers. Instead of improving patient privacy rights, increased administrative work and excessive technology requirements to ensure security may delay research projects and increase costs. Ultimately, the regulatory burden may turn against its objectives and impede progress in patient care [13].

## Data Availability

The source data and codes are available at https://github.com/obruck/Nav-Reg-Res.
